# Concentration and Distribution of Toxic and Essential Elements in Traditional Rice Varieties of Sri Lanka Grown on an Anuradhapura District Farm

**DOI:** 10.1007/s12011-023-03847-1

**Published:** 2023-09-19

**Authors:** Thomas E. Lockwood, Richard B. Banati, Chandima Nikagolla, Jake P. Violi, David P. Bishop

**Affiliations:** 1 Hyphenated Mass Spectrometry Laboratory (HyMaS), Faculty of Science, University of Technology Sydney, Ultimo, NSW 2007 Australia; 2 Australian Nuclear Science and Technology Organisation (ANSTO), Lucas Heights, NSW 2234 Australia; 3 Faculty of Medicine and Health, University of Sydney, Camperdown, NSW 2006 Australia; 4 Faculty of Engineering, School of Civil and Environmental Engineering, Queensland University of Technology, QLD, Brisbane, 4000 Australia; 5 School of Chemistry, University of New South Wales, Kensington, NSW 2052 Australia

**Keywords:** LA-ICP-MS, Sri Lankan rice varieties, Toxic heavy metals, Essential elements

## Abstract

Toxic heavy metals have been the focus of many investigations into chronic kidney disease of unknown aetiology (CKDu) within Sri Lanka. It has been hypothesised that exposure to nephrotoxic arsenic, cadmium and lead could play a role in the development of CKDu, and these metals have previously been found in unsafe concentrations in Sri Lankan rice. Traditional varieties of Sri Lankan rice remain popular due to their perceived health benefits, but their uptake of trace and toxic heavy metals remained unexplored. Here, we report a one-time, cross-sectional dataset on the concentrations of essential and toxic elements present in eleven samples of polished and unpolished traditional rice varieties, all regularly grown and sold in the Anuradhapura district, a CKDu hotspot. All rice was sourced from the same farm, with the exception of one store bought sample grown on another, unidentified farm. Cadmium concentrations varied significantly between varieties, and potentially unsafe concentrations of cadmium were detected in the store-bought sample (Suwadel, 113±13 μg kg^−1^). Elemental imaging of the grains revealed lead to be stored mainly in the rice bran, which is removed during polishing, while cadmium was distributed in the edible portion of the grain. Essential elements were generally higher in the traditional rice varieties than those reported for non-traditional varieties and are a potential source of trace elements for nutrient-deficient communities. The concentration of selenium, an element that plays a protective role in the kidneys, was too low to provide the minimum recommended intake. The methods developed in this study could be applied to a more comprehensive study of elemental uptake of rice under controlled growing conditions.

**Supplementary Information:**

The online version contains supplementary material available at 10.1007/s12011-023-03847-1.

## Introduction

Toxic heavy metals are found naturally throughout many different environments although typically at low concentrations and not in bio-available forms. Agricultural practices and activities including emissions from mining or fossil fuels, such as in power generation, release toxic heavy metals into the surrounding environment where they can then enter the local food web and bioaccumulate, increasing at higher trophic levels. Accumulation in food products grown in or near polluted areas is a major concern for human health. Toxic metal exposure is suspected to cause disease and may lead or contribute to the formation of sporadic neurodegenerative diseases, cancer and hepatic and nephrotic disorders [1]. Chronic kidney disease (CKD) has been linked with toxic metal exposure as arsenic, cadmium, chromium and lead are all known nephrotoxins that may cause kidney damage [2, 3].

Prevalence of CKD is high in Sri Lanka, with up to 12% of the population in the northern district of Anuradhapura affected. Of these cases, approximately 50% could not be linked to known risk factors and were defined as CKD of unknown aetiology (CKDu) [4]. CKDu is not associated with typical CKD risk factors such as diabetes or hypertension, and primarily affects young to middle age people living in low-income agricultural communities [5]. The cause of CKDu is unknown but likely multifactorial, with dehydration, genetics and exposure to environmental contaminants all playing a role [6]. Toxic heavy metals have been the focus of many investigations into CKDu in Sri Lanka, with elevated levels found in rice, groundwater and soil [7, 8].

Rice is the staple food for the majority of Sri Lankans with 50% of meals containing rice or rice derivatives [9]. To meet the demand for rice production, high-yield and pest-resistant rice varieties were developed, with the Sri Lankan Department of Agriculture introducing over 80 new rice varieties since the 1950s. While improved rice accounts for 99% of cultivated rice paddies, there are over 2000 traditional varieties still reported in Sri Lanka. Several hundred of these varieties are popular with farmers due to their nutritional value, adaptability and resistance to pests [10, 11].

When rice is grown in soil or irrigation water that is contaminated with toxic heavy metals, they are taken up by the plants roots and transported to the grain. The levels of As, Cd, Hg and Pb found in the grain correlate with those found in the soil [12]. Testing of rice paddy soils in Sri Lanka has shown typical levels of toxic heavy metals to be lower than those reported in other regions of the world, although higher concentrations are found in CKDu hotspots. Soils in both the wet and dry zones of Sri Lanka show enriched As, Cd and Pb from anthropogenic processes [8]. The primary source of heavy metal contamination is suspected to be the overuse of contaminated fertilisers [13]. Inorganic phosphorus fertilisers are used extensively by farmers seeking higher crop yields, and these same fertilisers have been shown to contain heavy metals [13, 14]. Environmental factors such as water hardness, soil pH and other types of pollution can also impact the uptake of heavy metals into plants, further increasing accumulation [15, 16].

Cadmium levels in Sri Lankan rice are the second highest globally [17]. Previous studies have found elevated levels of As, Cd and Pb in rice cultivated in regions endemic for CKDu [9, 17–19]. However, a recent study of Sri Lankan rice found similar concentrations of these metals in both endemic and non-endemic regions, with none exceeding the World Health Organizations (WHO) Codex standards [14]. Kulathunga et al. found that 23% of rice samples from CKDu patients had levels of Pb above WHO permissible limits [20]. It should be noted that these limits do not necessarily account for rice constituting such a high proportion of the Sri Lankan diet. Rice represents a major route for toxic metal contaminants to enter the body, and rice consumption has previously been linked with the outbreak of CKDu [17, 19, 21]. Studies of CKDu patients have found contradicting evidence of a direct link between As, Cd or Pb and CKDu pathogenesis [22, 23].

The uptake and distribution of essential elements and toxic heavy metals within rice are species-dependant and change the exposure risk to humans. The traditional rice varieties found in Sri Lanka have nearly twice the As and half the Cd of improved rice [9]. Storage of metals within individual rice grains also varies. Some species have been shown to accumulate Hg primarily within the scutellum while others distributed Hg within the endosperm, the edible portion of the grain [24]. This varied distribution of elements can affect the risk of toxic metal exposure via consumption. Most rice is polished, a process that discards the rice bran and germ. Both the bran and germ have relatively high metal contents and polishing removes 36% of Pb, 44% of As, 10–30% Cd and a significant fraction of essential trace metals [25–27]. Traditional red Sri Lankan rice varieties are generally only partially polished to retain the red colour, a product of their high anthocyanin content, potentially increasing their Pb and As consumption risk [11]. Although polishing can remove toxic heavy metals, it also increases their bioavailability and may not reduce the overall exposure risk [27].

Rice is the major dietary source of essential trace elements for Sri Lankans. Concentrations of essential elements in rice vary with the time of year, location and variety, but are typically lower than those of other cereals [28]. Nutrient deficiency is common in Sri Lanka, with one half of the population Fe or Zn deficient [29]. Insufficient intake of Fe, Cu and Zn can lead to a range of blood, gastrointestinal, nervous and immune disorders [30]. Selenium is an essential trace element that plays an important role in protecting the kidneys from oxidative stress caused by toxic heavy metals [31]. Se is used as a cofactor in the synthesis of glutathione peroxidases (GSH-Px) by the kidneys and low serum Se and GSH-Px are frequently found in individuals with CKD [32, 33]. Deficiency due to dietary intake is a common problem worldwide, particularly in areas with low protein intake [34]. Se is transported into rice and from the soil, and the depleted soils of Sri Lanka cause rice grown there to have some of the lowest Se levels in the region [9, 14, 34]. Se deficiency is common in Sri Lanka and has been observed in CKDu patients [22, 35]. The traditional rice varieties of Sri Lanka generally have higher concentrations of essential elements than improved rice due to enhanced elemental uptake by their relatively deep and branched root systems [36]. This is echoed in wild rice varieties from other parts of the globe, and these rice varieties could serve an improved dietary source of essential elements [37].

To date, there has been limited research into the uptake of metals by individual species of native rice. Previous studies have pooled samples into native and improved cohorts [9], or have only looked at improved rice [14]. It remains to be seen if species dependant uptake of nephrotoxic metals can help explain the large variation in concentrations observed, which range over an order of magnitude. This study determines both the concentration and distribution of toxic and essential elements in seven traditional rice varieties grown at a single farm in the Anuradhapura district. The data was examined to identify if traditional rice could be a possible route of nephrotoxic metal exposure or an improved source of trace elements for deficient communities.

## Methods

### Chemicals and Reagents

All ultra-pure water (18.2 MΩ cm^−1^) used was provided by a Sartorius™ Arium pro UF Ultrapure Water System. High purity liquid elemental standards and ultrapure nitric and hydrochloric acids (Seastar Baseline; 67–70% HNO_3_, 30–35% HCl) were all provided by Choice Analytical, Thornleigh, NSW, Australia. Selleys Araldite Ultra Clear Epoxy was used for mounting samples. 0.2-μm Captiva polyethersulfane syringe filters used were provided by Agilent Technologies, Mulgrave, Victoria, Australia. Plastic tubes used throughout experiments were made of polypropylene, with all pipette tips used being metal free (Gilson, WI, USA).

### Samples

Eleven samples of seven Sri Lankan traditional rice varieties (Suwadel, Kalu Heenati, Rathal, Madathwalu, Siyapathal (Pachchaperumal), Swanjatha, Ran Kahawanu) were obtained from a traditional rice farm in Medawachchiya, a high CKDu prevalence region within the Anuradhapura district of the North Central Province. These varieties represent some of the most popular traditional rice varieties among farmers and consumers in Sri Lanka [10]. With the exception of a store bought Suwadel sample, all rice was grown in the same rice farm, using the same geological water source. Rice varieties were grown in different seasons, depending on their growth time. Historical use of inorganic fertiliser at the farm could not be ruled out and may contribute to toxic metal concentrations. Both unhusked and polished samples of Suwadel, Kalu Heenati, Madathwalu and Ran Kahawanu were collected; the same mill was used for all polishing.

### Sample Preparation

For solution inductively coupled plasma-mass spectrometery (ICP-MS) analysis, 10 to 20 grains of each rice sample were ground to a fine powder in a ceramic mortar and pestle and passed through a 1-mm stainless steel mesh to remove the husk. Approximately 100 mg of the ground rice was added into three pre-weighed 10-ml polypropylene tubes along with 1 mL of HNO_3_ and 1 mL of HCl, providing three sample replicates. After an overnight digest, the samples were made up to 10 mL using ultrapure water and filtered using a 0.2-μm polyethersulfane filter. Only one replicate of Swanjatha could be performed due to the small quantity of sample available.

Elemental imaging of rice was performed by mounting grains to microscope slides using epoxy. Once the epoxy had hardened, the top third of each grain was removed using a razor. Grains were then ground and polished using 400 and 1200 grit lens polishing paper (Agilent Technologies) to reveal the cross-section of each rice grain and ensure a smooth and flat surface for imaging.

### Instrumentation

#### Solution ICP-MS

Acid-digested rice samples were analysed using an Agilent Technologies 7700s ICP-MS. Samples were introduced via a 1.02-mm internal diameter Tygon tubing connected to a three-channel peristatic pump. The samples and the internal standard, a solution of 100 ng mL^−1^ Rhodium in 1% HNO_3_, were combined in a post pump T connector. The combined solution was delivered to the ICP-MS via a MicroMist nebuliser and Scott type double pass spray chamber, cooled to 2 °C. Samples were continuously delivered to the ICP-MS at a flow rate of 0.1 mL min^−1^ for 1 min to allow for signal stabilisation, followed by a 30-s wash of 2% HNO_3_. The ICP-MS parameters are detailed in Table 1. Agilent Technologies ICP-MS Chemstation software was used for all instrument control and data analysis. Quantification was performed using an eight-point mixed element calibration curve from 0.6 to 600 μg kg^−1^. Linearity (*r*^2^) was greater than 0.9996 for all elements, and all samples were above the limits of detection.

**Table 1:** ICP-MS parameters for solution and laser ablation modes

Parameter	Solution	Laser	
Instrument	7700	7900	
RF power	1550	1350	W
Carrier gas flow rate	0.80	1.15	L min^−1^
Makeup gas flow rate	0.40	0.0	L min^−1^
Sample depth	8.0	5.0	mm
Extract 1, 2	−3.3, −200	−2.0, −240	V
Omega bias, lens	−120, 18.0	−115, 11.5	V
Cell entrance, exit	−50, −90	−50, −100	V
Octopole bias, RF	200, −20	200, −20	V
Deflect	2.0	2.8	V
He flow	4.4	0.0	mL min^−1^
H flow	0.0	3.0	mL min^−1^

#### LA-ICP-MS

Elemental distributions of epoxy embedded rice were collected using a New Wave Research NWR193 ArF excimer laser connected to an Agilent Technologies 7900 ICP-MS fitted with S lenses. Prior to running, the setup was tuned using NIST612–trace elements in glass. The laser was run with a fluence of 1.8 mJ cm^−2^, spot size of 50 μm, scan speed of 200 μm s^−1^ and frequency of 40 Hz. Lines were spaced at 50-μm intervals. A single low-power pre-ablation pass (50 μm, 400 μm s^−1^, 40 Hz) was used to clear the sample of any debris left by the polishing process. ICP-MS parameters are listed in Table 1. Elemental dwell times were set to multiples of 25 ms to reduce aliasing, and a total acquisition time of 0.25 s was used, producing square pixels. Images were processed using Pew^2^, and backgrounds were removed using watershed segmentation of the ^31^P image [38]. Quantification of the images was not performed due to a lack of appropriate standards. The images are presented with no interpolation of the elemental data.

## Results and Discussion

The variation and distribution of both toxic and essential trace elements in seven traditional rice varieties were measured using solution nebulisation ICP-MS and LA-ICP-MS. Two white varieties, Suwandel and Ran Kahawanu, were selected along with four red, Kalu Heenai, Rathel, Madathwalu and Siyapathal (Pachchaperumal). A small amount of husk-less red rice Swanjatha was also obtained. Samples of unpolished and polished rice (labelled P) were used to determine the effect polishing has on elemental concentrations.

### Nephrotoxic Metals

Nephrotoxic metal concentrations of the 11 rice samples are listed in Table 2. One sample of Suwadel rice exceeded the Australian guideline for Cd in rice (100 μg kg^−1^). No other sample exceeded the Australian, European Union or WHO guidelines for maximum levels of Cd in rice or As and Pb in cereals (1000 and 200 μg kg^−1^). Concentrations of Cr and Ni were not sufficient to exceed TDIs set by the European Food Safety Authority, assuming an average 60-kg body weight and consumption of 0.3 kg day^−1^ of rice [39, 40]. These levels are consistent with previous studies of Sri Lankan rice, which range from 20 to 200, 10 to 870 and 10 to 1800 μg kg^−1^ for As, Cd and Pb respectively [9, 14, 20]. This large range in concentrations may be due to a combination of the local conditions where the rice is sourced [9] and the differing uptake of metals by rice varietals [24]. Indeed, the two varieties of native white rice had very different Cd concentrations, 80 ± 50 and 23 ± 4 μg kg^−1^, and Kalu Heenati red rice was far below the mean red Cd concentration, 4 ± 2 versus the mean of 14 ± 10 μg kg^−1^. Unpolished red varietals had higher mean Pb (22 versus 6.8 μg kg^−1^) and lower Cd (12 versus 42 μg kg^−1^) concentrations than white rice, while As concentrations were similar between the two rice types. The differences in concentrations observed in this study are potentially a result of differential uptake of the metals as the growing conditions of all rice varieties were similar. Certain rice varieties may pose a greater health risk than others when grown in contaminated regions, and selection of varieties with lower toxic metal uptake could mitigate these risks. Suwadel is a highly popular traditional rice varietal and appears to have increased uptake of cadmium into the edible portion of the grain [26]. The historical usage of cadmium-contaminated inorganic fertilisers could not be ruled out, and metal concentrations vary spatially, even on the same farm [41], so a further study under more controlled conditions is required to see if Suwadel’s uptake rate is of concern. With an increasing number of farmers in Anuradhapura choosing to grow and consume traditional rice [10], and some of the highest CKDu incidences in the country [4], it is imperative to know which varieties are safe to cultivate in contaminated soils.

**Table 2:** Mean concentrations and standard deviations of nephrotoxic elements in rice (μg kg^*−1*^). *N*=3 except where noted, (P) denotes polished rice

Variety	Cr	Co	Ni	As	Cd	Pb
Suwadel	215 ± 69	55 ± 11	449 ± 82	45 ± 24^a^	45 ± 5	6.4 ± 1.4
Suwadel (P)	24 ± 25	18 ± 3	367 ± 40	57.8 ± 0.3	113 ± 13	3.0 ± 1.6
Kalu Heenati	25 ± 21	97 ± 33	410 ± 112	45 ± 31	2.4 ± 0.3	11 ± 4
Kalu Heenati (P)	49 ± 5	54 ± 9	671 ± 119	39 ± 2	5.6 ± 1.0	3.5 ± 0.5
Rathal	37 ± 7	36.6 ± 0.7	689 ± 39	49 ± 4	16.3 ± 1.3	15.5 ± 1.5
Madathwalu	619 ± 519	45 ± 3	580 ± 214	113 ± 32	19 ± 3	82 ± 22
Madathwalu (P)	42 ± 21	49 ± 8	757 ± 112	52 ± 5	7.8 ± 1.7	5.6 ± 0.4
Siyapathal	31 ± 14	32 ± 6	331 ± 86	47 ± 16	30 ± 7	6 ± 2
Swanjatha	121^b^	9^b^	306^b^	293^b^	4^b^	33^b^
Ran Kahawanu	251 ± 68	23 ± 4^a^	182 ± 63	90 ± 10	25 ± 3	15 ± 3
Ran Kahawanu (P)	65 ± 27	17.7 ± 1.8	163 ± 19	76 ± 12	20.1 ± 1.8	5.5 ± 1.9

^a^*N*=2, outlier removed due to failed *Q* test

^b^*N*=1, limited sample quantity

The limited number of samples and single location of the current study makes its application to the rest of Sri Lanka or even the Anuradhapura region speculative. Despite this, the results reveal that there may be species-dependant uptake of toxic heavy metals, and this deserves further investigation. There is currently little to no information about the uptake of metals in Sri Lankan traditional rice by individual varietals. The popularity of the selected varieties and general increased interest in traditional rice mean that any differential uptake in toxic heavy metals could have a large health impact.

**Fig. 1:**
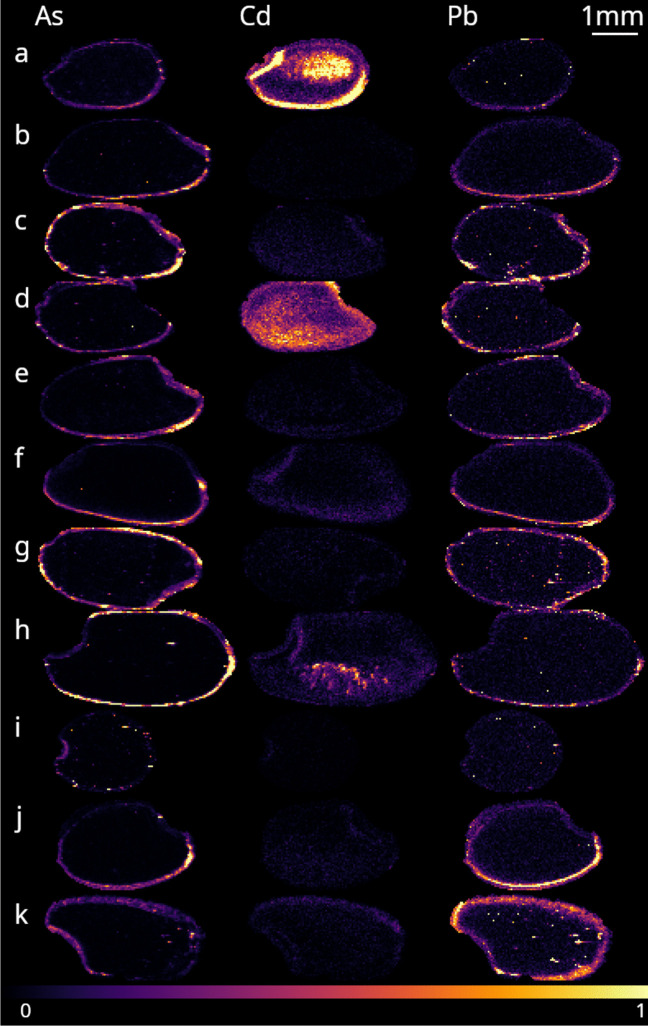
Toxic heavy metals in Sri Lankan traditional rice. Concentration and distribution of the metals varied with variety. From top to bottom: Suwadel (**a**), Kalu Heenati (**b**), Rathal (**c**), polished Suwadel (**d**), polished Kalu Heenati (**e**), Madathawalu (**f**), polished Madathawalu (**g**), Siyapathal (**h**), Swanjatha (**i**), Ran Kahawanu (**j**) and polished Ran Kahawanu (**k**). Colour-scales are in normalised counts for each element

LA-ICP-MS imaging reveals that the toxic heavy metals are stored differently in the rice grains (see Fig. 1). In most varietals, the Cd is distributed in the scutellum and throughout the grain while As and Pb are confined to the bran and germ. Polished rice had 52–93% lower Pb and lower As with the exception of Suwadel; no such trend was observed for Cd. This is consistent with previous studies that show most metals other than Ag and Cd are at least several times higher in the bran [42]. Distributions of all elements, micrographs and photographs of the rice are available in Figures S1 and S2.

### Essential Metals

Native varieties of Sri Lankan rice typically had higher concentrations of essential trace elements than rice grown elsewhere (Table 3, Table S1). Concentrations of Mn, Cu and Zn were from one to three times that of Australian and one to four times that of Bangladeshi rice [43]. Concentrations of Mn, Cu and Zn were higher than previously reported values in improved Sri Lankan rice [9]. Only Cu and Mn were significantly different (Welch’s *t* test; *p*=0.011, 0.016) between unpolished red and white rice, with increased Cu in red and increase Mn in white rice. Swanjatha, an ancient variety of husk-less red rice, contained the highest concentration of Mg, K, Ca, Al, Fe, Cu and Zn. These results suggest that traditional varieties of Sri Lankan rice, like other wild rice, could be a good source of essential elements [37].

**Table 3:** Essential metal concentrations and standard deviations in the Sri Lankan rice (mg kg^−*1*^). *N*=3 except where noted, (P) denotes polished rice

Variety	Mn	Fe	Cu	Zn	Se
Suwadel	38 ± 9	12 ± 2	2.7 ± 0.4	36.6 ± 1.9	0.089 ±0.014
Suwadel (P)	21 ± 3	4.1 ± 0.6	2.5 ± 0.3	25 ±3	0.116 ± 0.012
Kalu Heenati	22 ± 7	13 ± 4	4.7 ± 1.4	34 ± 7	0.07 ± 0.03
Kalu Heenati (P)	17 ± 3	10.5 ± 1.5	4.6 ± 0.8	29 ±2	0.104 ± 0.018
Rathal	16.5 ± 1.0	12.0 ± 1.0	2.76 ± 0.16	29 ± 4	0.109 ± 0.002
Madathwalu	22 ± 4	16 ± 2	4.94 ± 0.14	60 ± 41	0.07 ± 0.02
Madathwalu (P)	17 ± 3	11.1 ±1.5	5.1 ± 0.9	45 ± 9	0.10 ± 0.02
Siyapathal	24 ± 5	11 ± 3	3.7 ± 0.8	36 ± 9	0.08 ± 0.02
Swanjatha	28^b^	59^b^	6^b^	98^b^	0.08^b^
Ran Kahawanu	44 ± 9	16 ± 2	2.9 ± 0.3	37 ± 4	0.079 ± 0.009
Ran Kahawanu (P)	14.8 ± 1.2	4.2 ± 0.4	2.6 ± 0.2	30 ± 4	0.087 ± 0.015

^b^*N*=1, limited sample quantity

**Fig. 2:**
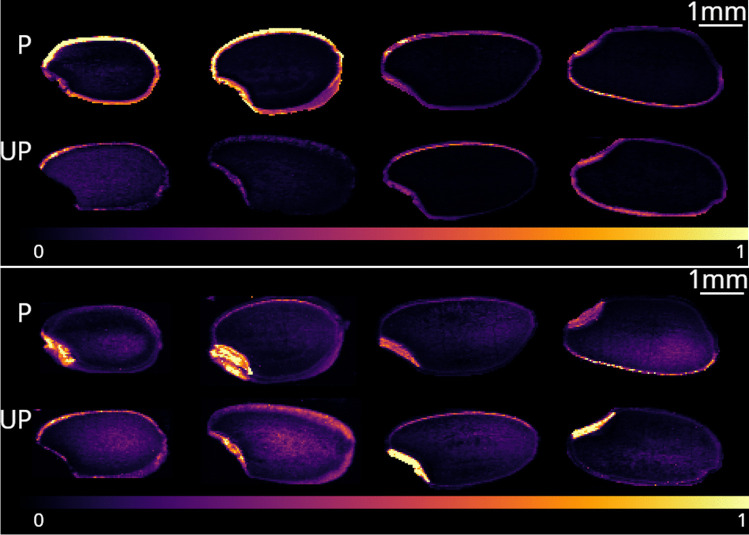
Manganese (top) and zinc in polished (P) and unpolished (UP) rice grains. Polishing removes the germ where many essential elements are stored, particularly in white rice. From left to right: Suwandel, Ran Kahawanu, Kalu Heenati and Madathawalu. Colour-scales are in normalised counts

Polished rice had lower concentrations of Cu and Mn, and significantly lower Fe and Zn (paired sample *t* test; *p*=0.046, 0.019). LA-ICP-MS imaging of Zn in Fig. 2 reveals that a large proportion of the element is stored in the germ of the rice. The distribution of Cu is similar, while Mn and Fe are concentrated primarily in the rice bran (Figure S1). Both the bran and germ are lost during polishing which explains the lower concentrations [25]. Consuming unpolished rice would increase essential element intake but also the intake of toxic heavy metals. A more gentle polishing regime, where only the bran is removed, could increase essential elements while still removing a large portion of As and Pb [44].

Selenium concentrations were similar across all rice varieties with a mean concentration of 0.090 ± 0.015 mg kg^−1^. This was higher than concentrations reported in previous studies of Sri Lankan rice (0.007–0.15 mg kg^−1^) and similar to those of Chinese (0.09 mg kg^−1^) and Indian (0.157 mg kg^−1^) rice [34]. Selenium intake from rice alone would contribute a maximum of 35 μg day^−1^, below the recommended daily intake of 55 μg [45]. While Se intake from consumption of native rice is higher than that of improved rice, it still cannot contribute the full amount required daily.

## Conclusions

The concentrations of nephrotoxic and essential elements were determined in eleven samples and seven traditional varieties of Sri Lankan rice. The concentration of Cd was above Australian guidelines in one sample; no other nephrotoxic metals were detected in unsafe concentrations. The large variation in elemental concentrations between the different rice varieties may either be the result of environmental variations or possibly be due to inherently different elemental uptake by the studied rice varieties, given that these were all, with one exception sample, grown in similar environments on the same organic rice farm. There remains uncertainty as to the historical use of inorganic fertiliser and small-scale spatial soil variations in the current study, so a future study with laboratory-controlled growing conditions would be required to confirm the differential uptake of elements. The data presented here is based on rice sourced from a small organic farm and thus should not be generalised to the wider Anuradhapura region, notably not to farms where rice varieties might be grown on heavily fertilised or contaminated soils. Concentrations of essential elements, including Se where higher than those of “improved” rice varieties, but were still insufficient to fulfil recommended dietary intakes. LA-ICP-MS was used to show elemental distributions within the grains and confirm why only specific elements are decreased during the polishing process.

### Supplementary Information

ESM 1 (PDF 657 kb)
