# Supplementary material for: Concentration and Distribution of Toxic and Essential Elements in Traditional Rice Varieties of Sri Lanka Grown on an Anuradhapura District Farm
Source: Biol Trace Elem Res. 2023 Sep 19;202(6):2891–9. doi: 10.1007/s12011-023-03847-1 (PMC11052878; doi:10.1007/s12011-023-03847-1)
Supplement: Supplementary file 1 — (PDF 657 kb) [file 12011_2023_3847_MOESM1_ESM.pdf]

Biological Trace Element Research

Supplementary Information for: Concentration and distribution of toxic and essential elements in traditional Sri Lankan rice varieties grown in the Anuradhapura district.

Thomas E. Lockwood, Richard B. Banati, Chandima Nikagolla, Jake P. Violi, David P. Bishop*

* Corresponding author: david.bishop@uts.edu.au

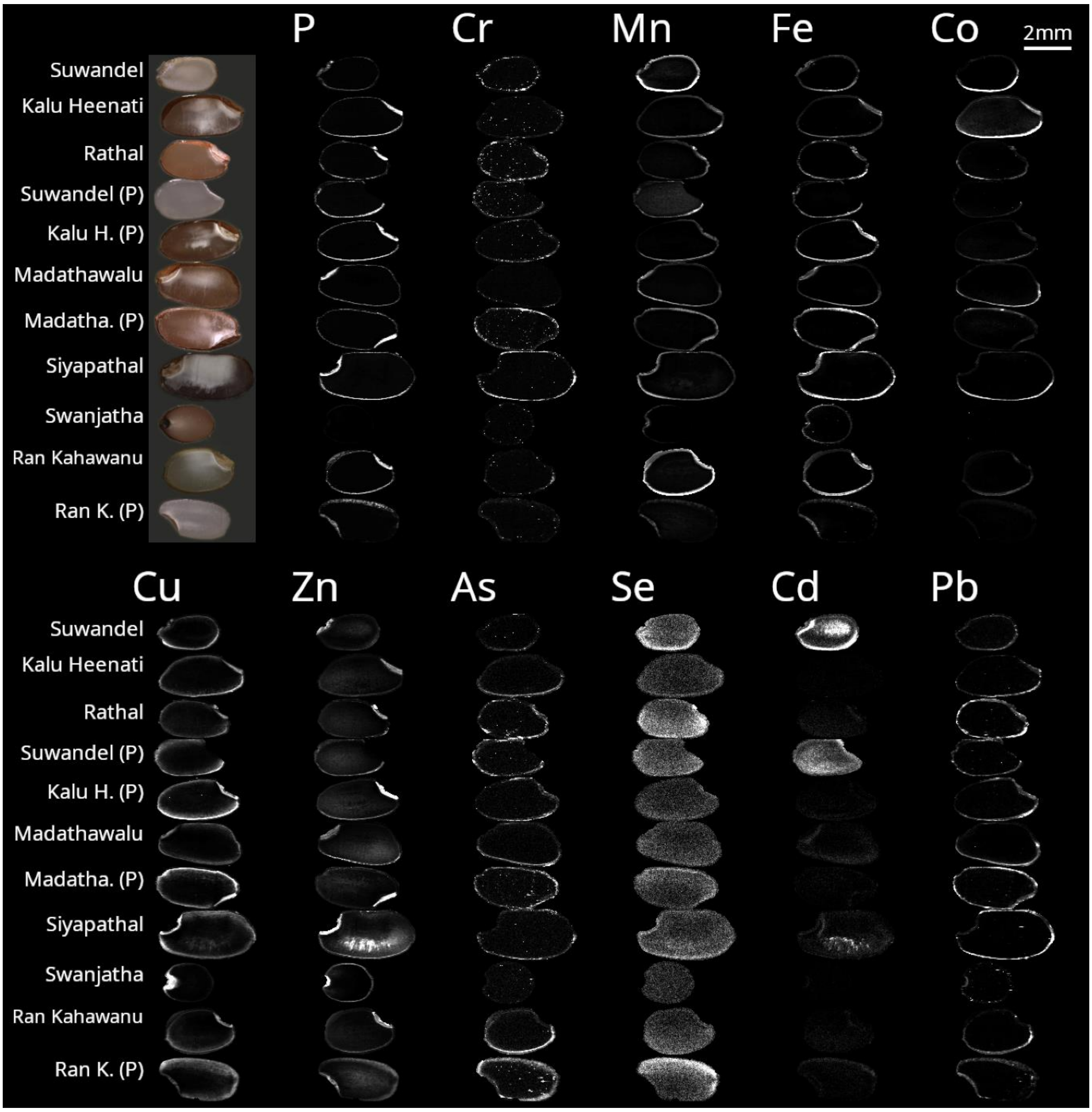

Figure S1. LA-ICP-MS images of various elements in traditional Sri Lankan rice grains. (P) = polished.

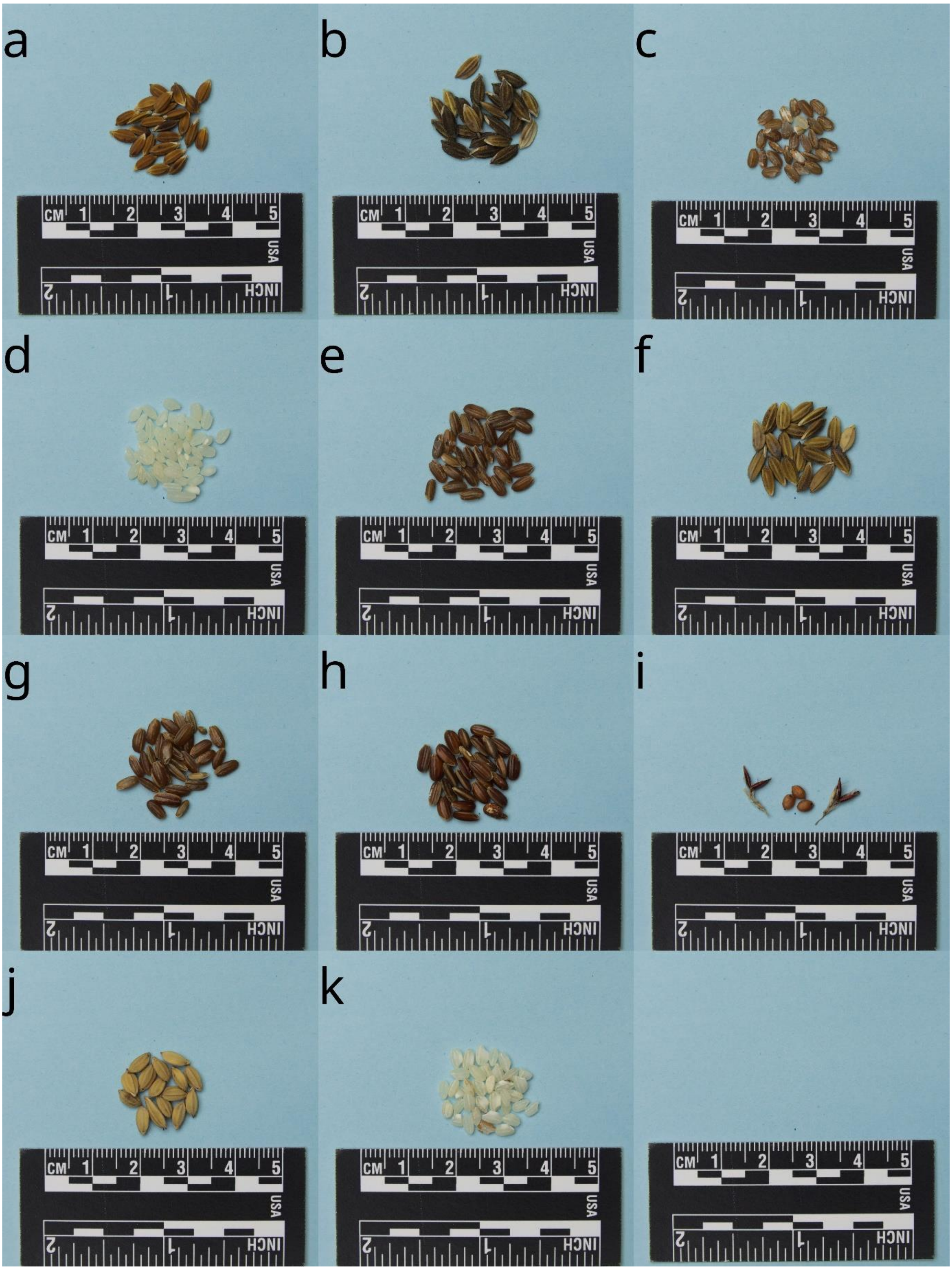

Figure S2. Photographs of the rice samples. (a) Suwandel, (b) Kalu Heenati, (c) Rathal, (d) Suwandel, (e) Kalu Heenati, (f) Madathawalu, (g) Madathuwalu, (h) Siyapathal, (i) Swanjatha, (j) Ran Kahawanu, (k) Ran Kahawanu

Table S1. Mean concentrations and standard deviations of elements in varieties of traditional Sri Lankan rice (mg kg^{-1}). N=3 except where noted, (P) = polished.

**Variety**	**Mg (x10^3)**	**Al**	**K (x10^3)**	**^{43}Ca**	**Ti**	**V**	**Cr**	**Mn**	**Fe**	**Co**
Suwadel	1.23 ± 0.05	0.77 ± 0.05 [a]	2.59 ± 0.15	21 ± 2	0.6 ± 0.2	0.5 ± 0.3	0.21 ± 0.07	38 ± 9	12 ± 2	0.055 ± 0.011
Suwadel (P)	0.671 ± 0.017	1.3 ± 0.3	1.58 ± 0.04	14.1 ± 0.9	0.45 ± 0.06	1.19 ± 0.17	0.02 ± 0.02	21 ± 3	4.1 ± 0.6	0.02 ± 0.03
Kalu Heenati	1.30 ± 0.06	1.6 ± 0.3	2.65 ± 0.17	18.3 ± 0.5	0.5 ± 0.2	0.8 ± 0.2	0.02 ± 0.02	22 ± 7	13 ± 4	1.0 ± 0.3
Kalu Heenati (P)	1.275 ± 0.019	0.9 ± 0.2	2.71 ± 0.06	16.1 ± 0.6	0.61 ± 0.11	0.78 ± 0.05	0.049 ± 0.005	17 ± 3	10.5 ± 1.5	0.054 ± 0.009
Rathal	1.43 ± 0.05	1.21 ± 0.06	3.12 ± 0.13	16.6 ± 1.1	0.66 ± 0.06	0.84 ± 0.09	0.037 ± 0.007	16.5 ± 1.0	12.0 ± 1.0	0.0366 ± 0.0007
Madathwalu	1.25 ± 0.08	2.99 ± 0.14	2.95 ± 0.16	22.5 ± 1.1	0.57 ± 0.10	1.3 ± 0.3	0.6 ± 0.5	22 ± 4	16 ± 2	0.045 ± 0.003
Madathwalu (P)	1.28 ± 0.09	1.3 ± 0.3	2.66 ± 0.16	17.9 ± 1.9	0.61 ± 0.12	1.22 ± 0.14	0.04 ± 0.02	17 ± 3	11.1 ±1.5	0.049 ± 0.008
Siyapathal	1.83 ± 0.10	1.5 ± 0.4	3.9 ± 0.2	21.6 ± 1.5	0.72 ± 0.18	0.96 ± 0.19	0.31 ± 0.14	24 ± 5	11 ± 3	0.032 ± 0.006
Swanjatha	2.5 [b]	23 [b]	5.1 [b]	47 [b]	0.58 [b]	0.68 [b]	0.12 [b]	28 [b]	59 [b]	0.10 [b]
Ran Kahawanu	1.12 ± 0.08	0.82 ± 0.12	2.7 ± 0.2	21.6 ± 1.5	0.54 ± 0.05	1.17 ± 0.10	0.25 ± 0.07	44 ± 9	16 ± 2	0.023 ± 0.004 [a]
Ran Kahawanu (P)	0.44 ± 0.03	1.94 ± 0.18	1.27 ± 0.05	13.4 ± 1.4	0.46 ± 0.07	0.89 ± 0.16	0.07 ± 0.03	14.8 ± 1.2	4.2 ± 0.4	0.018 ± 0.005

**Variety**	**Ni**	**Cu**	**Zn**	**As**	**Se**	**Mo**	**Cd (x10^{-3})**	**Pb (x10^{-3})**	**Bi (x10^{-3})**	**U (x10^{-3})**
Suwadel	0.45 ± 0.08	2.7 ± 0.4	36.6 ± 1.9	0.05 ± 0.02 [a]	0.089 ± 0.014	0.36 ± 0.07	45 ± 5	6.4 ± 1.4	17.80 ± 0.17 [a]	0.12 ± 0.05
Suwadel (P)	0.37 ± 0.04	2.5 ± 0.3	25 ± 3	0.058 ± 0.003 [a]	0.116 ± 0.012	0.45 ± 0.05	113 ± 13	3.0 ± 1.6	32 ± 3	0.001 ± 0.011
Kalu Heenati	0.41 ± 0.11	4.7 ± 1.4	34 ± 7	0.05 ± 0.03	0.07 ± 0.03	0.39 ± 0.13	2.4 ± 0.3	11 ± 4	68 ± 11	0.16 ± 0.08
Kalu Heenati (P)	0.67 ± 0.12	4.6 ± 0.8	29 ± 2	0.039 ± 0.002	0.104 ± 0.018	0.41 ± 0.06	5.6 ± 1.0	3.5 ± 0.5	83 ± 14	0.09 ± 0.04
Rathal	0.69 ± 0.04	2.76 ± 0.16	29 ± 4	0.049 ± 0.004	0.109 ± 0.002	0.45 ± 0.03	16.3 ± 1.3	15.5 ± 1.5	69 ± 3	0.137 ± 0.015
Madathwalu	0.6 ± 0.2	4.94 ± 0.14	60 ± 41	0.11 ± 0.03	0.07 ± 0.02	0.31 ± 0.05	19 ± 3	80 ± 20	37 ± 3	0.37 ± 0.08
Madathwalu (P)	0.76 ± 0.11	5.1 ± 0.9	45 ± 9	0.05 ± 0.03	0.10 ± 0.02	0.36 ± 0.07	7.8 ± 1.7	5.6 ± 0.4	72 ± 16	0.08 ± 0.04
Siyapathal	0.33 ± 0.09	3.7 ± 0.8	36 ± 9	0.047 ± 0.005	0.08 ± 0.02	0.40 ± 0.08	30 ± 7	6 ± 2	110 ± 50	0.10 ± 0.07
Swanjatha	1.3 [b]	6 [b]	98 [b]	0.3 [b]	0.08 [b]	0.45 [b]	4 [b]	33 [b]	73 [b]	n.d.
Ran Kahawanu	0.31 ± 0.06	2.9 ± 0.3	37 ± 4	0.090 ± 0.010	0.079 ± 0.009	0.42 ± 0.05	25 ± 3	15 ± 3	24 ± 2	0.140 ± 0.005
Ran Kahawanu (P)	0.182 ± 0.019	2.6 ± 0.2	30 ± 4	0.076 ± 0.011	0.087 ± 0.015	0.42 ± 0.05	20.1 ± 1.8	5.5 ± 1.9	65 ± 6	0.049 ± 0.017

[a] N=2, outlier removed as failed Q-test

[b] N=1, limited sample quantity
